# JMJD6 promotes melanoma carcinogenesis through regulation of the alternative splicing of PAK1, a key MAPK signaling component

**DOI:** 10.1186/s12943-017-0744-2

**Published:** 2017-11-29

**Authors:** Xujun Liu, Wenzhe Si, Xinhua Liu, Lin He, Jie Ren, Ziran Yang, Jianguo Yang, Wanjin Li, Shumeng Liu, Fei Pei, Xiaohan Yang, Luyang Sun

**Affiliations:** 1 Key Laboratory of Carcinogenesis and Translational Research (Ministry of Education), Department of Biochemistry and Molecular Biology, School of Basic Medical Sciences, Peking University Health Science Center, Beijing, 100191 China; 2 Department of Laboratory Medicine, Peking University Third Hospital, Beijing, 100191 China; 3 Department of Biochemistry and Molecular Biology, School of Basic Medical Sciences, Tianjin Medical University, Tianjin, 300070 China; 4 Department of Pathology, School of Basic Medical Sciences, Peking University Health Science Center, Beijing, 100191 China; 5 Department of Biochemistry and Molecular Biology, Peking University Health Science Center, 38 Xueyuan Road, Beijing, 100191 China

**Keywords:** JMJD6, PAK1, MAPK, Melanoma, Alternative splicing

## Abstract

**Background:**

Melanoma, originated from melanocytes located on the basal membrane of the epithelial tissue, is the most aggressive form of skin cancer that accounts for 75% of skin cancer-related death. Although it is believed that *BRAF* mutation and the mitogen-activated protein kinase (MAPK) pathway play critical roles in the pathogenesis of melanoma, how the MAPK signaling is regulated in melanoma carcinogenesis is still not fully understood.

**Methods:**

We characterized JMJD6 expression in melanoma tissue array by immunohistochemistry analysis. We used human melanoma A375, 451Lu and SK-MEL-1 cell lines for in vitro proliferation and invasion experiments, and xenograft transplanted mice using murine melanoma B16F10 cells by bioluminescence imaging for in vivo tumor growth and pulmonary metastasis assessments. Endothelial tube formation assay, chicken yolk sac membrane assay and matrigel plug assay were performed to test the effect of JMJD6 on the angiogenic potential in vitro and in vivo*.*

**Results:**

Here we report that the jumonji C domain-containing demethylase/hydroxylase JMJD6 is markedly up-regulated in melanoma. We found that high expression of JMJD6 is closely correlated with advanced clinicopathologic stage, aggressiveness, and poor prognosis of melanoma. RNA-seq showed that knockdown of JMJD6 affects the alternative splicing of a panel of transcripts including that encoding for PAK1, a key component in MAPK signaling pathway. We demonstrated that JMJD6 enhances the MAPK signaling and promotes multiple cellular processes including melanogenesis, proliferation, invasion, and angiogenesis in melanoma cells. Interestingly, JMJD6 is transcriptionally activated by c-Jun, generating a feedforward loop to drive the development and progression of melanoma.

**Conclusions:**

Our results indicate that JMJD6 is critically involved in melanoma carcinogenesis, supporting the pursuit of JMJD6 as a potential biomarker for melanoma aggressiveness and a target for melanoma intervention.

**Electronic supplementary material:**

The online version of this article (10.1186/s12943-017-0744-2) contains supplementary material, which is available to authorized users.

## Background

Melanoma is the most aggressive form of skin cancer, with a global incidence of approximately 200,000 new cases per year. Although it represents only 4% of all skin cancers, melanoma is correlated with approximately 80% of skin cancer-related deaths [1]. Survival rates depend on the clinical stage at the diagnosis, with 5-year survival ranging from 15% to 60% in patients with distant and local metastases [2].

Approximately 50% of melanomas harbor an active mutation in BRAF, most commonly BRAF^V600E^ mutation [3]. RAF kinases are components of the mitogen-activated protein kinase (MAPK) pathway. BRAF^V600E^ mutation renders the kinase constitutively active and entails enhanced growth and invasion of melanoma cells [4]. The MAPK pathway is initiated by receptor tyrosine kinases (RTK), cytokine receptors, or G proteins, which activate a class of intracellular protein serine/threonine kinases including RAF [5], which dimerizes and phosphorylates/activates MEK1/2, which in turn phosphorylates and activates ERK1/2. The MAPK signaling cascade leads to the phosphorylation of c-Jun, resulting in transcriptional activation of cyclin D1 [6], which triggers melanocyte proliferation and angiogenesis [7]. Moreover, MAPK can also influence other cellular processes including apoptosis [8] and cell migration and invasion [9, 10]. However, the regulatory mechanism of the MAPK signaling pathway itself and the impact of such regulation on melanoma carcinogenesis require further exploration.

JMJD6 was originally identified as a membrane protein and a putative receptor for phosphatidylserine acting in phagocytosis [11]. Subsequent investigations including studies by our own lab indicate that this jumonji C domain-containing protein also functions in the nucleus where it acts as a histone arginine demethylase or lysyl oxidase to target histones [12] or non-histone proteins including the tumor suppressor p53 [13]. Interestingly, a more recent study showed that JMJD6 catalyzes lysyl-5-hydroxylation of the splicing factor U2AF65, leading to the alteration of alternative RNA splicing of a set of the endogenous genes [14]. *Jmjd6* ablation in mice results in early postnatal lethality, growth retardation and multiple developmental abnormalities due to impaired differentiation during embryogenesis [15–17]. These findings support a multifaceted and important role for JMJD6 in cell biology and animal development. Correspondingly, JMJD6 has been implicated in various pathological states including cancers [13, 18, 19]. However, a functional role of JMJD6 in melanoma remains to be explored.

Alternative splicing is a process by which different combinations of exons can be joined together to produce multiple mRNA isoforms from a single transcript, generating proteins differing in structure, function, and localization [20]. In humans, >95% of multi-exonic protein-coding genes undergo alternative splicing [21]. Given that alternative splicing plays a key role in the regulation of gene expression, aberrant splicing has thus been implicated in a wide range of human diseases [22]. Alterations in alternative splicing are commonly reported in various cancers with involving genes exemplified by p53 and PTEN [23], BRCA1 [24], and PRMT2 [25] in breast cancer, TIMP1 and CD44 in colon cancer [26], Bcl-xL and CD44 [27] in lung cancer, and calpain 3 in melanoma [28]. As a prominent etiological factor, whether or not the MAPK signaling is regulated via alternative splicing in melanoma is currently unknown.

In the current study, we found that JMJD6 is markedly up-regulated in melanoma, and that high expression of JMJD6 is closely correlated with advanced clinicopathologic stage, aggressiveness, and poor prognosis of melanoma. We showed that JMJD6 regulates the alternative splicing of a collection of transcripts including that encoding for PAK1, a key component in the MAPK signaling pathway. We showed that JMJD6 positively regulates the MAPK signaling and promotes the melanogenesis, proliferation, angiogenesis, and invasion of melonama cells. We demonstrated that JMJD6 is transcriptionally activated by c-Jun, generating a feedforward loop to drive the development and progression of melanoma.

## Methods

### Cell cultures

Human melanoma A375, SK-MEL-1 cell lines and murine melanoma B16F10 cell line were obtained from the American Tissue Culture Collection (ATCC). Human melanoma 451Lu cell line was obtained from the Beijing Cancer Hospital. Cells were cultured in Dulbecco’s Modified Eagle Medium (HyClone) supplemented with 1% penicillin-streptomycin and 10% fetal bovine serum (FBS). HUVECs were cultured in Endothelial Cell Medium (ECM, ScienCell) supplemented with 1% penicillin-streptomycin and 1% endothelial cell growth factors. Cells were incubated at 37 °C in a CO_2_ incubator with a humidified atmosphere containing 5% CO_2_.

### Transfection

The A375 and 451Lu cells were grown in a 6-well plate to almost 70%-80% confluence, and transfected with 2.5 μg empty vector (pCMV-Tag 2B), FLAG-JMJD6, FLAG-JMJD6H187A/D189A (JMJD6m), FLAG-c-Jun, FLAG-PAK1 or FLAG-PAK1Δ15 plasmids using PEI reagent (Polysciences) according to manufacturer’s instructions. JMJD6m was generated by using QuikChange Lightning Site-Directed Mutagenesis Kit (Agilent). The A375 and 451Lu cells were grown in a 6-well plate to almost 30%-40% confluence, siRNA oligonucleotides were transfected into cells using RNAiMAX (Invitrogen) with the final concentration at 25 nM. The sequences of siRNAs were: siJMJD6#1, 5′-GAGGGAACCAGCAAGACGA-3′; siJMJD6#2, 5′-GUGUGGUGAGGAUAACGAU-3′; siBRAF#1, 5′-CAUGAAGACCUCACAGUAA-3′; siBRAF#2, 5′-UCAGUAAGGUACGGAGUAA-3′; siBRAF#3, 5′-AGACGGGACUCGAGUGAUG-3′; sic-Jun#1, 5′-GAUGGAAACGACCUUCUAU-3′; sic-Jun#2, 5′-CUGAUAAUCCAGUCCAGCA-3′ and siControl, 5′-UUCUCCGAACGUGUCACGU-3′. All of the siRNAs were synthesized by GeneChem Inc. (Shanghai, China).

### Retroviral and Lentiviral production and infection

The retroviral plasmid vector, pBABE-JMJD6, pBABE-JMJD6m, pBABE-PAK1, or pBABE-PAK1Δ15, together with pVSV-G and pGag-Pol were co-transfected into the packaging cell line 293T. Viral supernatants were collected 48 h later, clarified by filtration, and concentrated by ultracentrifugation. Lentiviruses carrying control shRNA (shControl), PAK1 shRNA (shPAK1), JMJD6 shRNAs (shJMJD6) and Jmjd6 shRNAs (shJmjd6) were purchased from Genepharma Inc. The virus was used to infect 5 × 10^5^ cells (30%-40% confluent) in a 6-cm dish with 8 μg/ml polybrene. Infected cells were selected by 5 μg/ml puromycin (Merck). The sequences of shRNAs were: shPAK1: 5′-CCGGGGTTTCAAGTGTTTAGTAACTCTCGAGAGTTACTAAACACTTGAAACCTTTTTG-3′, shJMJD6#1: 5′-CCGGGGAAAGGGCAGATGCTTTACACTCGAGTGTAAAGCATCTGCCCTTTCCTTTTTG-3′, shJMJD6#2: 5′-CCGGGGTGGCATGTTGTCCTCAATCCTCGAGGATTGAGGACAACATGCCACCTTTTTG-3′; shJmjd6#1: 5′- CCGGGGAGAGAGCTGATGCCTTACACTCGAGTGTAAGGCATCAGCTCTCTCCTTTTTG -3′; shJmjd6#2: 5′-CCGGGCGTTCTGGAACTGGGATTCACTCGAGTGAATCCCAGTTCCAGAACGCTTTTTG-3′ and shControl: 5′-CCGGGAATCGTCGTATGCAGTGAAACTCGAGTTTCACTGCATACGACGATTCTTTTTG-3′.

### Immunohistochemical analysis

Malignant melanoma microarray, containing 128 cases of primary malignant melanoma and 64 metastatic malignant melanoma, was purchased from US Biomax (Catalog number: ME2082b). Antigen retrieval was performed by incubating the samples in 0.01 M sodium citrate buffer at high pressure. Subsequently, the samples were blocked in 10% normal goat serum in PBS and then incubated with primary antibody solution containing anti-JMJD6 (1:100) at 4 °C overnight. After washing with 0.01 M PBS buffer, the samples were incubated with HRP-conjugated goat anti-mouse secondary antibodies for 30 min at 25 °C, developed with DAB (3,3′-diaminobenzidine tetrahydrochloride), and counterstained with hematoxylin. All specimens were examined by two pathologists who were blinded to the clinical data. In case of discrepancies, a final score was established by reassessment on a double-headed microscope. In scoring JMJD6 expression, both the intensity and extent of immunopositivity were considered. Intensity of immunopositivity was scored as follows: 0, negative; 1, weak; 2, moderate; and 3, strong. Extent of immunopositivity was quantified according to the percentage of positively stained tumor cells: 0, <5%; 1, >5%-25%; 2, >25%-50%; 3, >50%-75%; and 4, >75%. The final score was determined by multiplying the intensity scores and the extent scores, which yielded a range from 0 to 12.

### Total RNA extraction and RT-PCR analysis

Total RNA was isolated from cells using TRIzol reagent (Invitrogen). First strand cDNA was synthesized using 1 μg of total RNA, with the EasyScript First-Strand cDNA Synthesis SuperMix (TransGen Biotech). Quantification of all gene transcripts was done by qPCR using Power SYBR Green PCR Master Mix (Roche) and an ABI PRISM 7500 sequence detection system (Applied Biosystems). *GAPDH* was used as the internal control. Primer sequences for human genes are as follows: *JMJD6* forward: 5′-AAACTTTTGGAAGACTACAAGGTGC-3′ and reverse: 5′-CCCAGAGGGTCGATGTGAATC-3′; *GAPDH* forward: 5′-CCCACTCCTCCACCTTTGAC-3′ and reverse: 5′-CATACCAGGAAATGAGCTTGACAA-3′ and *MITF* forward: 5′-CCACCAAGTACCACATACAG-3′ and reverse: 5′-ACATCATCCATCTGCATACAG-3′. Primer sequences for mouse genes are as follows: *Jmjd6* forward: 5′-GGAGATATTACAGAAACCAGGAG-3′ and reverse: 5′-CTCCTGTTTCAAGATCCTATACC-3′; and *Gapdh* forward: 5′-GACAACTTTGGCATTGTGGA-3′ and reverse: 5′-CATCATACTTGGCAGGTTTCTC-3′. *PAK1* forward: 5′-CACCAATGGGACCCCAGAACTT-3′ and reverse: 5′-GCAGTTCTCTTCAATGCTGGACACA-3′ for splice-specific RT-PCR. For quantification of PAK1 and PAK1*Δ*15 mRNA expressions, TaqMan assays were performed using TaqMan *PAK1* probes or *PAK1Δ15* probes together with PAK1 specific primers. *PAK1* TaqMan probe: 5′-FAM-TGCTACAGGTGAGAAAACTG-MGB-3′, *PAK1Δ15* TaqMan probe: 5′-FAM-TGCTGCTACAGCATCAATTC-MGB-3′, *PAK1* forward: 5′-GAAAACCCTCTGAGAGCCTTGTACC-3′ and reverse: 5′-ATCAGTGGAGTGAGGCTGGAGA-3′ for TaqMan assays. Each independent experiment was performed at least three times.

### RNA-seq analysis

In brief, A375 cells were treated with control siRNA or JMJD6 siRNAs. After transfection for 48 h, total RNA was isolated from the cells. For RNA-seq, mRNA-Seq Sample Prep Kit (Illumina) was used to prepare the complementary DNA (cDNA) libraries according to the manufacturer’s protocol. After selecting cDNA fragments of approximately 200 bp by agarose gel electrophoresis, the DNA fragments were ligated with Illumina paired-end sequencing adapters and performed with PCR amplification. Finally, Illumina HiSeqTM 2000 system was used to perform RNA-seq.

### Western blotting analysis

Cells were lysed with RIPA buffer (1% NP40, 50 mM Tris-HCl (pH 7.4), 1 mM EDTA, and 150 mM NaCl) containing a protease inhibitor cocktail. The BCA Protein Assay Kit (Thermo Fisher Scientific) was used to determine the protein concentration. Equal amounts of protein were resolved using 10% SDS-PAGE gels and transferred onto polyvinylidene fluoride (PVDF) membranes (Roche). The PVDF membranes were blocked with 5% skim milk for 1 h at room temperature. Subsequently, PVDF membranes were incubated with the appropriate antibodies overnight at 4 °C. After washed with PBST solution the membranes were incubated with secondary antibodies for 1 h at room temperature. Western blotting luminol reagent (Santa Cruz) was used to visualize the immunoreactive bands according to the manufacturer’s protocol.

### RNA Immunoprecipitation (RIP) assay

10^7^ A375 cells were harvested and resuspended in 2 ml PBS, 2 ml nuclear isolation buffer (40 mM Tris-HCl pH 7.5, 4% Triton X-100, 1.28 M sucrose, 20 mM MgCl_2_), and 6 ml water on ice for 30 min with frequent mixing. Nuclei were pelleted by centrifugation at 3000 g for 10 min. Nuclear pellet was resuspended in 1 ml RIP buffer (150 mM KCl, 25 mM Tris pH 7.4, 9 μg/ml leupeptin, 5 mM EDTA, 9 μg/ml pepstatin, 10 μg/ml chymostatin, 0.5% NP40, 3 μg/ml aprotinin, 1 mM PMSF, 0.5 mM DTT, 100 U/ml RNase inhibitor). Resuspended nuclei were mechanically sheared using a dounce homogenizer with 20 strokes. Nuclear membrane and debris were pelleted by centrifugation at 13000 g for 10 min. The supernatant was split into three fractions for IP (JMJD6 or IgG) and input. 5 μg anti-JMJD6 antibody (mouse monoclonal, sc-28348; Santa Cruz) or 5 μg anti-mouse IgG (sc-2025; Santa Cruz) was added to IP fractions, and was incubated for 2 h at 4 °C with gentle rotation. 40 μl protein G beads were added and incubated for 1 h at 4 °C with gentle rotation. Beads were pelleted at 2000 g for 1 min, the supernatant was removed, and beads were washed with RIP buffer three times, followed by one wash in PBS. Beads were resuspended in 1 ml of TRIzol, and bound RNA was then detected by qRT-PCR. The primers were the following: PAK1#1 forward: 5′-AAATAACACCACTCCACCAG-3′ and reverse: 5′-AAGCCTACGACCAGCAAATC-3′; and PAK1#2 forward: 5′-ATTGGACAAGGGTTAGTAGG-3′ and reverse: 5′-GTAATTTGGGAAGTTGGTTT-3′.

### Measurement of melanin content

B16F10 cells were infected with viruses and subsequently were treated with α-MSH (100 nM) for 18-24 h. The cells were washed with PBS solution and dissolved in 1 M NaOH containing 10% DMSO for 1 h at 90 °C. The melanin content was measured at 475 nm using VARIOSKAN FLASH (Thermo Fisher Scientific). To accurately measure the melanin content in each sample, the melanin measurement was normalized to the protein concentration. Each independent experiment was performed at least three times.

### Colony formation assay

Colony formation assay was used to determine the function of JMJD6 in cell proliferation. In brief, A375 cells were infected with viruses carrying JMJD6 shRNA, and/or PAK1, PAK1Δ15, JMJD6, or JMJD6m. Then, 5 × 10^3^ cells were seeded onto 6-well culture plates. After incubation for 14 days, cells were washed three times with cold phosphate-buffered saline (PBS) solution and fixed with 4% paraformaldehyde. Finally, the colonies were stained with 0.5% crystal violet solution. Colonies were counted using a light microscope. Each independent experiment was performed at least three times.

### Cell proliferation analysis

The cell viability rate of A375 was evaluated using Cell Counting Kit-8 (CCK-8 Kit, Beyotime Institute of Biotechnology). A375 cells were infected, and a total of 2500 cells were seeded onto 96-well plates with 200 μl of DMEM. Every 12 h, the cells were treated with 20 μl of CCK-8 solution and incubated for another 30 min. The absorbance of each well was measured at 450 nm using VARIOSKAN FLASH (Thermo Fisher Scientific). The blank controls contained 20 μl of CCK-8 solution in 200 μl of DMEM. The blank absorbance was subtracted, and the cell proliferation curve was drawn. Each independent experiment was performed at least three times.

### Transwell assay

In vitro invasion assay was performed using Transwell migration chambers (8 μm pore size, BD Biosciences). The filters were coated with 100 μl Matrigel (BD Biosciences) and incubated in a 5% CO_2_ atmosphere at 37 °C for 30 min for gelling. The bottom chambers were filled with 500 μl DMEM medium containing 10% FBS. A total of 2 × 10^4^ cells were resuspended in 100 μl DMEM serum-free medium and seeded in the upper chamber, and then incubated at 37 °C with 5% CO_2_ atmosphere for 12 h. Cells were then fixed with 4% paraformaldehyde and stained with 0.5% crystal violet for 20 min. The top surface of the membrane was gently scrubbed with a pipette tip or cotton tipped applicator, the cells that had migrated to the lower side were counted under the microscope and the numbers of migrated cells were calculated as the mean ± standard deviation (SD) in 10 different fields of view. Each independent experiment was performed at least three times.

### Bioluminescence imaging

For bioluminescence imaging, each mouse was first anesthetized and then given 150 μg/g of D-luciferin in PBS by intraperitoneal injection. After 20 min, bioluminescence images were obtained with a charge-coupled device camera (IVIS; Xenogen). Bioluminescence was calculated manually from the relative optical intensity, and the data were expressed as photon flux (photons·sec^−1^·cm^−2^·Steradian^−1^) and normalized to background photon flux, which was defined as the relative optical intensity of a mouse that was not injected with luciferin.

### Subcutaneous tumor growth model and pulmonary metastasis model

B16F10 cells that were infected with lentiviruses carrying control shRNA or Jmjd6 shRNAs were collected and washed three times with cold PBS solution. Cellular viability was assessed by staining the cells in 0.4% trypan blue followed by hemocytometer quantification according to the manufacturer’s protocol and was standardized at a minimum of 95% viability for subsequent experiments. For subcutaneous tumor growth analysis, 3 × 10^5^ live cells were injected subcutaneously on the right flank of C57BL/6 mice (*n* = 6). The tumor volume was measured every 2 days and calculated by the following formula: tumor volume = width^2^ × length × π/6. For pulmonary metastasis analysis, 5 × 10^5^ live cells were intravenously injected into C57BL/6 mice (*n* = 6) via the tail vein. All mice were euthanized, and the tumors were removed.

### Endothelial tube formation assay

Angiogenesis was detected in vitro using HUVECs and Basement Membrane Matrix (BD Biosciences). A total of 200 μl of Basement Membrane Matrix was coated on each well of the 24-well cell culture plates, and the Basement Membrane Matrix was polymerized at 37 °C for at least 1 h. HUVECs (5 × 10^4^ cells/well) infected with viruses carrying JMJD6 shRNA, or PAK1 shRNA, and/or PAK1, PAK1Δ15, JMJD6m, or cultured with CM from A375 cells that were infected with viruses carrying corresponding plasmids were added onto the solidified extracellular matrix gel in 700 μl of ECM, and were treated with or without 20 ng/ml VEGF (Abcam). After 6-18 h of incubation, the number of endothelial cell tubes was quantified under a light microscope. Each independent experiment was performed at least three times.

### Chicken yolk sac membrane (YSM) assay

Fertilized eggs were incubated at 38 °C and cracked on day 3. On day 8, gelatin sponges were cut to a size of 1 mm^3^ and placed on top of the YSM under sterile conditions. The gelatin sponges were presoaked in A375 cell suspensions (approximately 3 × 10^5^ cells/sponge) that were infected with viruses carrying JMJD6 shRNA, and/or PAK1, PAK1Δ15, JMJD6, or JMJD6m. On day 12, the vessels of the YSM were detected and counted under a light microscope. Each independent experiment was performed at least three times.

### Matrigel plug assay

The Matrigel plug analysis was performed as previously described [29]. Briefly, 200 μl of matrigel only (BD Biosciences) or matrigel that was mixed with A375 cells (2 × 10^5^ cells) that were infected with lentiviruses carrying control shRNA or JMJD6 shRNA were subcutaneously injected into the left flank of 6-week-old BALB/c female mice (*n* = 6). Seven days after injection, all mice were sacrificed and the Matrigel plugs were retrieved; then, the Matrigel plugs were fixed with 4% paraformaldehyde and stained with Masson’s trichrome and H&E. Each independent experiment was performed at least three times.

### Luciferase reporter analysis

The promoter region (−1057 to +100) of *JMJD6* was cloned into pGL3 plasmid. The QuikChange Lightning Site-Directed Mutagenesis kit (Agilent Technologies, USA) was used to mutate the promoter region of *JMJD6.* A375 cells were transfected with JMJD6-Luc or Mut-JMJD6-Luc, together with c-Jun and renilla. 24 h after transfection, the luciferase activity of the cells was detected using a luciferase assay kit (Promega). Each independent experiment was performed at least three times.

### ChIP and qChIP analysis

ChIP assays were performed in A375 cells. The enrichment of the DNA template was analyzed by conventional PCR using the following primers: forward: 5′-CAAGAAGGGGAAAGCCCAGAT-3′ and reverse: 5′-TGTTGGGAAGGTCACGTCG-3′, which were specific for the *JMJD6* gene promoter. qChIP analysis was performed using the TransStart Top Green qPCR supermix (TransGen Biotech). The qChIP primers were the following: *JMJD6*#1 forward: 5′-CTACAGGCACAAGCCACGAT-3′ and reverse: 5′-GGGACCAGGAGTTCCAGACC-3′; and *JMJD6*#2 forward: 5′-CGCGCAGAACTGGCAAC-3′ and reverse: 5′-TACTCCTTCACATACGGCGG-3′. Each independent experiment was performed at least three times.

### Statistical analyses

All data were analyzed using SPSS 18.0. All data were shown as the mean ± SD, unless declared, and each independent experiment was performed at least three times. Paired-sample *t* tests based on a bi-directional hypothesis for continuous variables were used to assess the comparisons between adjacent normal tissue and cancer tissue. The various clinicopathological characteristics of JMJD6 expression were examined by the chi-square test in 88 melanoma specimens of melanoma microarray for whom the complete information on age, sex, pathological characteristics and TNM staging is available. The relationship between JMJD6 expression and the clinical staging was assessed by two-tailed unpaired *t* test in these 88 cases. *p < 0.05 was considered statistically significant.

## Results

### JMJD6 is up-regulated in melanoma, and high expression of JMJD6 protein is strongly correlated with advanced stages and aggressiveness of melanoma

**Fig. 1:**
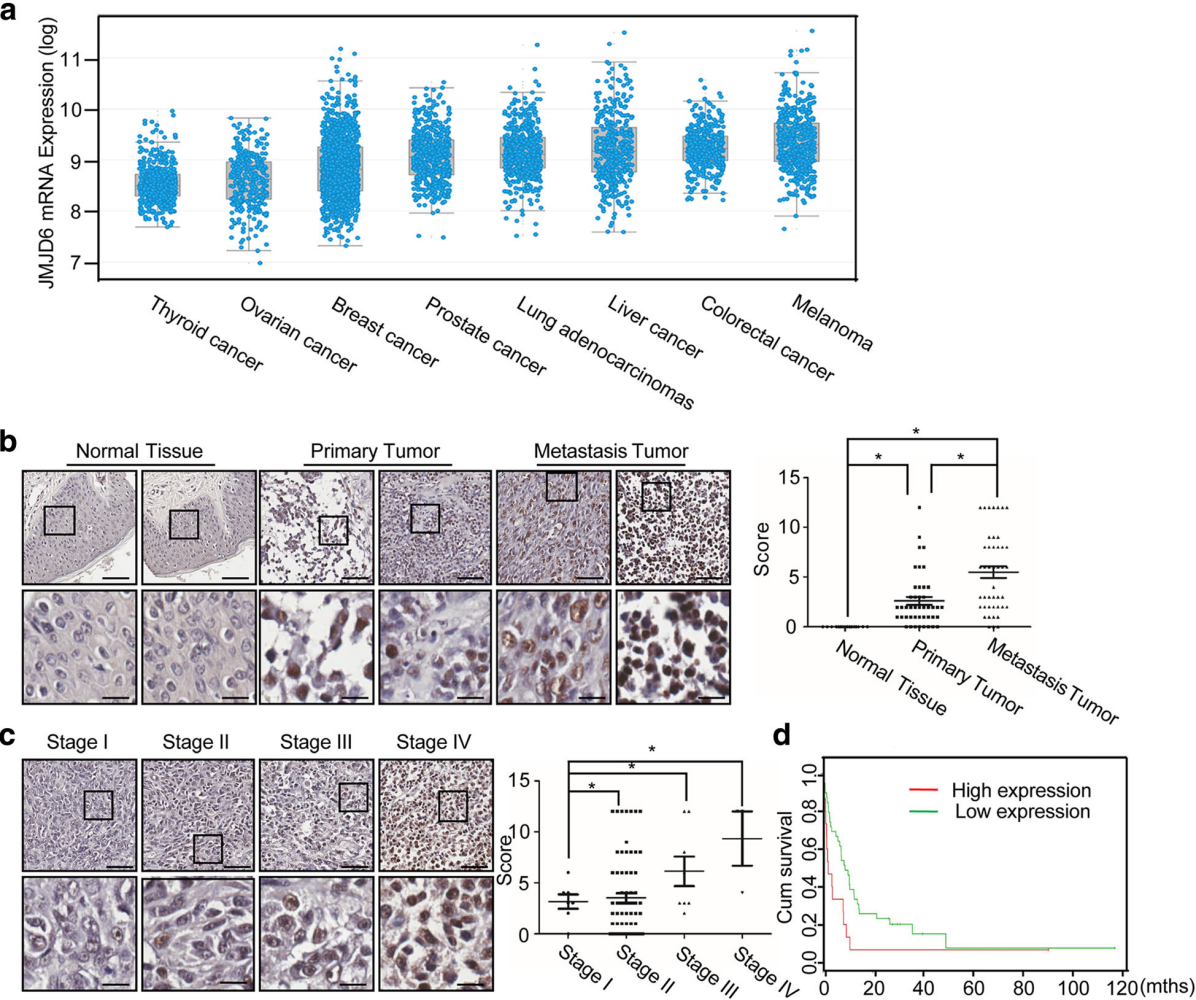
JMJD6 Is Up-regulated in Melanoma, and High Expression of JMJD6 Is Correlated with Melanoma Aggressiveness. **a** JMJD6 mRNA expression levels in multiple cancers by online analysis (www.cbioportal.org/public-portal/). **b** Representative sections of normal, primary melanomas and metastatic melanomas that were stained with JMJD6 antibody are presented (Upper magnification: × 20; scale bar = 100 μm. Lower magnification: × 320; scale bar = 25 μm). The scores were determined by evaluating the intensity and extent of immunopositivity. The results were analyzed by two-tailed unpaired *t*-tests (*p < 0.05). **c** Representative sections of clinicopathologic stages I, II, III and IV of melanoma that were stained with JMJD6 antibody are presented (Upper magnification: × 20; scale bar = 100 μm. Lower magnification: × 320; scale bar = 25 μm). The scores were determined by evaluating the intensity and extent of immunopositivity. The results were analyzed by two-tailed unpaired *t*-tests (*p < 0.05). **d** The survival rate of melanoma patients with high or low JMJD6 expression was analyzed by the Mantel-Cox log-rank test (*p < 0.05) (http://bioinformatica.mty.itesm.mx:8080/Biomatec/SurvivaX.jsp)

To gain more mechanistic insights into the role of JMJD6 in carcinogenesis, we analyzed the expression of JMJD6 across a set of cancer types on the website for cBioPortal for Cancer Genomics [30]. The analysis revealed that the mRNA level of JMJD6 was up-regulated in a variety of cancers, including thyroid cancer, ovarian cancer, breast cancer, prostate cancer, lung adenocarcinomas, liver cancer, colorectal cancer and melanoma (Fig. 1a). JMJD6 has been reported to play an important role in breast cancer [18] and lung adenocarcinomas [19, 31], and our previous study indicates that JMJD6 is critically involved in colon carcinogenesis [13]. To investigate the role of JMJD6 in melanoma carcinogenesis, we first detected JMJD6 expression in melanomas by tissue array. A series of carcinoma and normal tissues from melanoma patients were collected, including 128 primary melanomas, 64 metastatic melanomas, and 16 normal tissues. Immunohistochemical staining revealed that JMJD6 protein was mainly detected in the nucleus of melanoma cells, and that JMJD6 expression was markedly up-regulated in both primary melanomas and metastatic melanomas, with higher expressions of JMJD6 detected in metastatic melanomas (Fig. 1b). The further analysis was performed in 88 melanoma patients for whom complete information on age, sex, pathological characteristics and TNM staging is available. Remarkably, the level of JMJD6 protein expression was correlated with clinicopathologic stages (Fig. 1c), and high expression of JMJD6 was closely correlated with lymph node metastasis and distant metastasis, while no correlations were found between JMJD6 expression and gender, age, or depth of invasion (Additional file 1: Table S1). Moreover, we found a significant negative correlation between JMJD6 expression and the survival rate of patients (Fig. 1d). Collectively, these findings suggest that JMJD6 plays an important role in the development and progression of melanoma.

### JMJD6 enhances the MAPK signaling in melanoma cells through regulation of alternative splicing

**Fig. 2:**
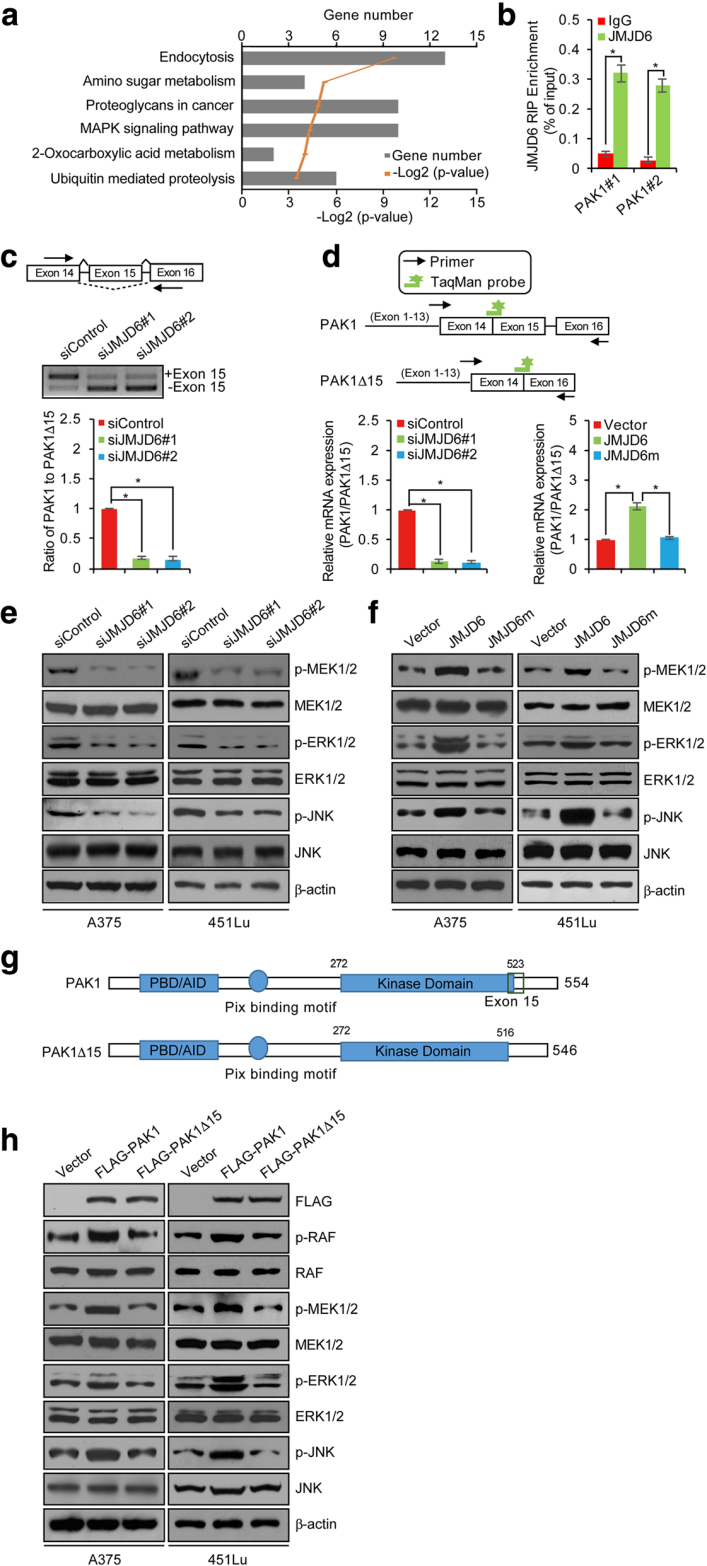
JMJD6 Enhances MAPK Signaling in Melanoma Cells through Regulation of Alternative Splicing. **a** Bioinformatics analysis of the different AS events that were identified in JMJD6-depleted A375 cells using the DAVID Functional Annotation Tool (DAVID, https://david.ncifcrf.gov/). **b** RNA-IP assay in A375 cells was performed with IgG or JMJD6 antibody followed by qRT-PCR with primer pairs for the intron-exon junction in PAK1 pre-mRNA. **c** The transcripts of full length (PAK1) and exon 15-skipped PAK1 (PAK1Δ15) were analyzed by RT-PCR using RNA extracted from control siRNA, siJMJD6#1 or siJMJD6#2-treated A375 cells. Arrows indicate the location of primers for RT-PCR analyses. Quantitation was done by densitometry and expressed as signals of PAK1/PAK1Δ15 ratios. **d** Schemes illustrating primer and probe design to detect PAK1 and PAK1Δ15 are shown. A375 cells were transfected with control siRNA/vector, siJMJD6#1 or siJMJD6#2, JMJD6, or JMJD6m, and TaqMan assays were performed to determine the ratio of PAK1 to PAK1Δ15 expression. **e, f** A375 and 451Lu cells were treated with control siRNA, siJMJD6#1, siJMJD6#2, vector, JMJD6 or JMJD6m, and Western blotting analysis was performed with antibodies as indicated. **g** A schematic representation of the structure of PAK1 and PAK1Δ15. The PBD (p21-binding domain), AID (auto inhibitory domain), and kinase domain are shown. The PAK1Δ15 is lack of 15th exon (517-533 aa) thus has an incomplete kinase domain. **h** A375 and 451Lu cells were transfected with vector, FLAG-PAK1 or FLAG-PAK1Δ15 expression plasmids, and Western blotting analysis was performed with antibodies as indicated

As JMJD6 has been reported to regulate RNA alternative splicing through hydroxylation of the alternative splicing regulator U2AF65 [14], to further explore the importance of JMJD6 in melanoma carcinogenesis and to understand the molecular mechanisms underlying the role of JMJD6 in the development and progression of melanoma, JMJD6 was knocked down in melanoma A375 cells using different JMJD6 siRNAs, and the influence of loss-of-function of JMJD6 on the global profile of the alternative splicing network was analyzed by RNA deep-sequencing (RNA-seq). With a *p*-value cutoff of <0.05, we identified 589 alternative splicing events that were significantly altered in the JMJD6-depleted cells. Notably, JMJD6 regulated the alternative splicing of genes that are related to several KEGG signaling pathways, including MAPK signaling pathway, endocytosis, amino sugar metabolism, proteoglycans in cancer, 2-oxocarboxylic acid metabolism, and ubiquitin-mediated proteolysis (Fig. 2a).

As stated above, the MAPK signaling pathway plays a critical role in melanoma carcinogenesis [6, 7]. Thus, the regulation of the MAPK signaling pathway by JMJD6 in melanoma cells supports a role of JMJD6 in the pathogenesis of melanoma. Bioinformatics analysis of alternative splice variants revealed that JMJD6 silencing affected the alternative splicing of several key components of the MAPK signaling pathway, including PAK1, RAPGEF2, and MAP3K4 (Additional file 2: Table S2). Among these, PAK1 is known to directly phosphorylates both RAF and MEK1, thereby positively regulating the MAPK signaling [32–34]. In addition, PAK1 is essential for oncogenic Ras- and ErbB2-regulated MAPK signaling and tumorigenesis [35]. Thus, the regulation of PAK1 alternative splicing by JMJD6 would have significant consequences on the MAPK signaling and cellular processes of melanoma cells.

To further investigate whether JMJD6 mediates PAK1 splicing, RNA-IP assay was performed with JMJD6 antibody followed by qRT-PCR with primer pairs for the intron-exon junction in *PAK1* pre-mRNA. The result showed that JMJD6 could bind to *PAK1* pre-mRNA, and the binding efficacy of JMJD6 to PAK1 was approximately 0.3% (Fig. 2b). We then designed primer pairs that were able to detect alternative exons. These primers were used to analyze RNAs isolated from control or JMJD6-depleted cells by reverse transcriptase (RT) PCR. We detected a decreased level of the full length PAK1 in JMJD6-depleted melanoma cells. Meanwhile, the level of the exon-skipped PAK1 mRNA isoforms, PAK1Δ15, was significantly elevated in JMJD6-depleted melanoma cells (Fig. 2c). Consistently, qRT-PCR measurement showed that JMJD6 knockdown led to a decrease in the production of the full length of PAK1 and an increase in the generation of PAK1Δ15 (Fig. 2d). In support of these observations, the catalytically inactive JMJD6 mutant, JMJD6H187A/D189A (JMJD6m), which can interfere with Fe(II) binding to JMJD6 and then lose its hydroxylase activity for U2AF65 and capacity for RNA splicing, was generated [14, 36]. The results showed that overexpression of JMJD6, but not JMJD6m, in melanoma A375 cells was associated with an increased level of the full length PAK1 mRNA and a decreased level of PAK1Δ15 mRNA (Fig. 2d). These results indicate that JMJD6 regulates the alternative splicing of PAK1 in melanoma cells by promoting exon inclusion and generation of the full length PAK1. These experiments also indicate that JMJD6 does so, through its lysyl hydroxylase activity.

In order to further support the observation that JMJD6 regulates the alternative splicing of PAK1 and to explore the functional significance of this regulation, the phosphorylation/activation of the PAK1 downstream kinases MEK, ERK, and JNK was analyzed. Western blotting showed that while the levels of the protein expression of these kinases did not change in JMJD6-depleted A375 and 451Lu cells, the levels of their phosphorylation were significantly reduced upon JMJD6 knockdown (Fig. 2e), suggesting that the activity of the MAPK signaling pathway was down-regulated by JMJD6 depletion.

To gain further support that JMJD6 modulates the MAPK signaling through its regulation of PAK1 alternative splicing, wild-type JMJD6 or JMJD6m was overexpressed in A375 or 451Lu cells. Western blotting analysis showed that overexpression of wild-type JMJD6, but not JMJD6m, was associated with a significant increase in the phosphorylation levels of MEK1/2, ERK1/2, and JNK, while neither wild-type JMJD6 nor JMJD6m affected the protein levels of MEK1/2, ERK1/2, and JNK (Fig. 2f). Collectively, the above experiments indicate that JMJD6, through regulating PAK1 alternative splicing, positively influences the MAPK signaling in melanoma cells, supporting the role of JMJD6 in melanoma carcinogenesis.

As stated above, PAK1 acts to promote MAPK pathway signaling through phosphorylating RAF and MEK [32, 33]. The kinase domain of PAK1 is mapped to 272-523 aa [37]. Interestingly, PAK1Δ15 is lack of the 15th exon (517-533 aa) thus has an incomplete kinase domain (272-516 aa) (Fig. 2g). To gain more insights into the functional significance of PAK1 alternative splicing, we firstly cloned FLAG-tagged PAK1 and PAK1Δ15 constructs, and examined their enzymatic activities. Western blotting analysis demonstrated that overexpression of PAK1, but not PAK1Δ15, was associated with a significant increase in the phosphorylation level of RAF, MEK1/2, ERK1/2, and JNK, although the total protein levels of RAF, MEK1/2, ERK1/2, and JNK were similar in cells transfected with PAK1 or PAK1Δ15 (Fig. 2h). These results suggest that PAK1Δ15, with its kinase domain interrupted, has a compromised kinase activity.

### JMJD6 promotes Melanogenesis in melanoma cells

**Fig. 3:**
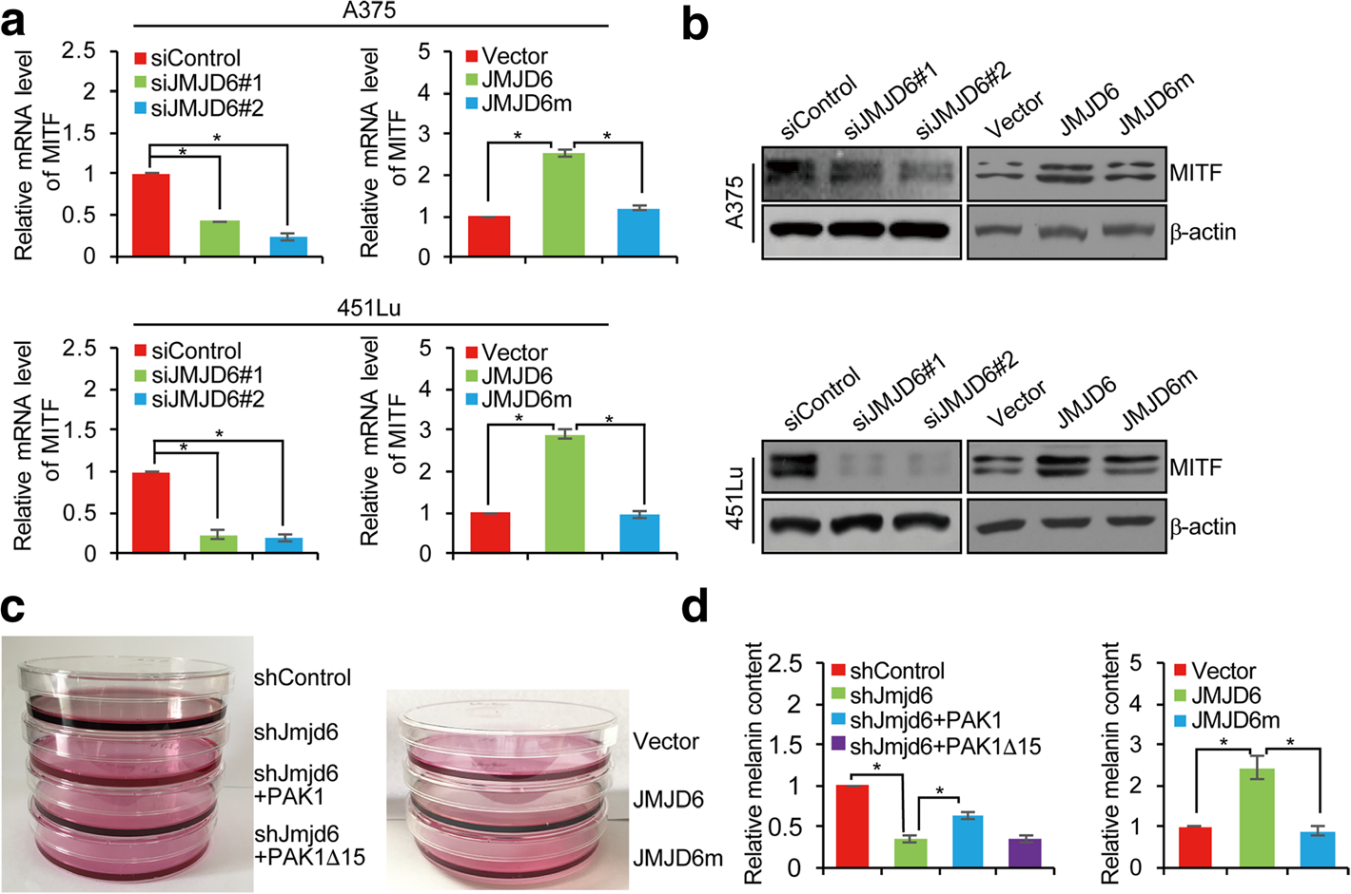
JMJD6 Promotes Melanogenesis in Melanoma Cells. **a** A375 and 451Lu cells were transfected with control siRNA/vector, siJMJD6#1, siJMJD6#2, JMJD6, or JMJD6m. Total RNAs were extracted and analyzed for the mRNA expression of MITF by qRT-PCR. Results were presented as the mean ± SD. **b** A375 and 451Lu cells were transfected with control siRNA/vector, siJMJD6#1, siJMJD6#2, JMJD6, or JMJD6m. Western blotting analysis was performed with antibodies against indicated proteins. **c** B16F10 cells were infected with lentiviruses carrying control shRNA or Jmjd6 shRNA, and/or infected with retroviruses carrying vector or expression plasmids for PAK1, PAK1Δ15, JMJD6, or JMJD6m, and treated with α-MSH (100 nM). The culture dishes of B16F10 cells were photographed. **d** The B16F10 cells infected with viruses carrying indicated constructs were incubated with α-MSH (100 nM) for 24 h. Melanin content was measured at 475 nm and normalized to the protein concentration. Each bar represents the mean ± SD. Each independent experiment was performed at least three times

Melanin synthesis is a unique characteristic of melanoma and a complex process involving microphthalmia-associated transcription factor (MITF), a transcription factor and a master regulator of melanogenesis, whose expression itself is activated by the MAPK signaling [38–40]. In order to understand the biological significance of the regulation of the MAPK signaling by JMJD6, we next investigated the effect of JMJD6 on melanogenesis. qRT-PCR and Western blotting analyses in A375 and 451Lu cells transfected with control siRNA/vector, JMJD6 siRNAs, JMJD6, or JMJD6m showed that both mRNA and protein levels of MITF were down-regulated when JMJD6 was knocked down. In agreement, overexpression of JMJD6 resulted in an increase in the expression of MITF, whereas overexpression of JMJD6m had a limited effect on MITF expression (Fig. 3a and b). These results suggest that JMJD6 might regulate melanogenesis in melanoma cells.

We then infected B16F10 cells, a melanin-producing mouse melanoma cell line, with lentiviruses carrying control shRNA or Jmjd6 shRNA, and/or infected with retroviruses carrying vector or expression plasmids for PAK1, PAK1Δ15, JMJD6, or JMJD6m. After treatment with α-MSH, the culture medium became grey when Jmjd6 was depleted, an effect that could be offset by simultaneous overexpression of PAK1, but not PAK1Δ15 (Fig. 3c). In contrast, the color of the culture medium was much darker upon overexpressing JMJD6, whereas overexpression of JMJD6m had no such an effect (Fig. 3c). Consistently, measurement of melanin content showed that cellular melanin level decreased in Jmjd6-depleted B16F10 cells, which could be rescued by simultaneous overexpression of PAK1, but not PAK1Δ15, whereas overexpression of JMJD6, but not JMJD6m, was associated with an increase in the cellular melanin level (Fig. 3d). Collectively, these results indicate that JMJD6 promotes melanogenesis, and that JMJD6 does so, through its lysyl hydroxylase activity and via its regulation of PAK1 alternative splicing.

### JMJD6 promotes the proliferation and invasion of melanoma cells in vitro and the growth and metastasis of melanoma in vivo

**Fig. 4:**
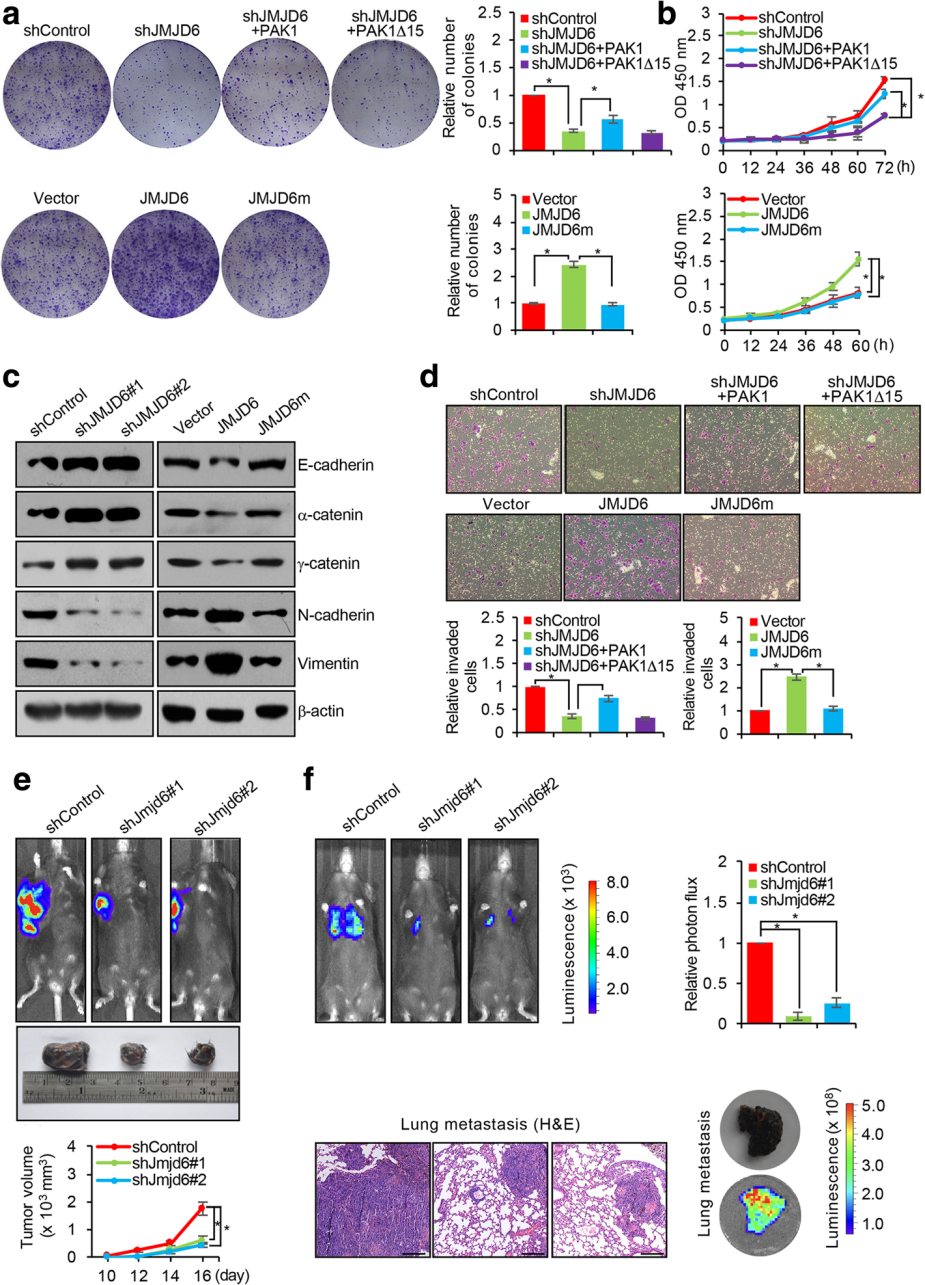
JMJD6 Promotes the Proliferation and Invasion of Melanoma Cells in Vitro and in Vivo*.* **a** A375 cells were infected with lentiviruses carrying control shRNA or JMJD6 shRNA, and/or infected with retroviruses carrying vector or expression plasmids for PAK1, PAK1Δ15, JMJD6, or JMJD6m. Cells were maintained in DMEM medium for 14 days before staining with 0.5% crystal violet and counting for colony numbers. **b** A375 cells were infected with viruses carrying indicated constructs, and the cell viability rate was evaluated using Cell Counting Kit-8. **c** A375 cells were infected with control shRNA/vector, JMJD6 shRNAs, JMJD6, or JMJD6m, and the expressions of epithelial and mesenchymal markers were tested by Western blotting. **d** The SK-MEL-1 cells infected with viruses carrying indicated constructs were starved for 18 h before cell invasion assays were performed using Matrigel transwell filters. The invaded cells were stained by 0.5% crystal violet and counted under a light microscope. **e** B16F10 cells were infected with viruses carrying control shRNA or Jmjd6 shRNAs. These cells were subcutaneously injected into the right flank of 6-week-old female C57BL/6 mice (*n* = 6). The bioluminescence imaging was used to quantify tumor size, and representative in vivo bioluminescent images are shown. **f** B16F10 cells were injected intravenously through the tail vein of 6-week-old female C57BL/6 mice (*n* = 6). Lung metastasis was quantified by bioluminescence imaging. Scale bar = 250 μm. Representative in vivo bioluminescent images are shown. Lung cancer specimens were examined by in vitro bioluminescent measurement and stained with hematoxylin and eosin (H&E)

To further explore the functional significance of the regulation of the alternative splicing of PAK1 by JMJD6 and to substantiate the role of JMJD6 in melanoma carcinogenesis, we next investigated the effect of JMJD6 on the proliferation of melanoma cells. To this end, A375 cells were infected with lentiviruses carrying control shRNA or JMJD6 shRNA, and/or infected with retroviruses carrying vector or expression plasmids for PAK1, PAK1Δ15, JMJD6, or JMJD6m. Colony formation assays showed that the depletion of JMJD6 in A375 cells resulted in a significant decrease in the colony number (Fig. 4a). Consistent with our working model that JMJD6 acts to regulate the alternative splicing of PAK1 by promoting exon inclusion to generate the full-length PAK1, simultaneous overexpression of PAK1, but not PAK1Δ15, led to a rescue of the effect of JMJD6-depleted on cell proliferation (Fig. 4a). In contrast, overexpression of JMJD6, but not JMJD6m, resulted in an increase in the colony number (Fig. 4a). Analogously, the growth curves determined by CCK-8 assays showed that JMJD6 depletion was associated with a significant decrease in cell proliferation, an effect that could be offset by ectopic expression of PAK1, but not PAK1Δ15, whereas overexpression of JMJD6, but not JMJD6m, was accompanied by a significant increase in cell proliferation (Fig. 4b). These results indicate that JMJD6 promotes melanoma cell proliferation, and that JMJD6 does so, through its lysyl hydroxylase activity and via regulating PAK1 alternative splicing.

The MAPK signaling has also been implicated in the regulation of epithelial-to-mesenchymal transition (EMT) [41], a hallmark of cancer that represents the initial step of tumor metastasis [42]. Given our observations that JMJD6 regulates the alternative splicing of PAK1 and influences the MAPK signaling in melanoma cells, it is conceivable that JMJD6 is also involved in the regulation of EMT and metastasis in melanoma. To test this, A375 cells were infected with viruses carrying control, JMJD6 shRNAs, JMJD6, or JMJD6m. Western blotting analysis indicate that JMJD6 depletion was associated with increased expression of epithelial markers E-cadherin, α-catenin, and γ-catenin and decreased expression of mesenchymal markers N-cadherin and vimentin. Consistently, overexpression of JMJD6, but not JMJD6m, was associated with decreased expression of the epithelial markers and increased expression of the mesenchymal markers (Fig. 4c). These data support a notion that JMJD6 promotes EMT in melanoma cells.

To substantiate this notion, transwell invasion assays showed that JMJD6 depletion resulted in a decrease in the invasive potential of the highly invasive SK-MEL-1 melanoma cells, an effect that could be offset by simultaneous overexpression of PAK1, but not PAK1Δ15, whereas overexpression of JMJD6, but not JMJD6m, led to an increase in the invasive potential of cells (Fig. 4d). Together, the above results support an argument that JMJD6 promotes the proliferation and invasion of melanoma cells in vitro, and that JMJD6 does so, through its lysyl hydroxylase activity and via regulating PAK1 alternative splicing.

To investigate the role of JMJD6 in melanoma growth and metastasis in vivo, murine B16F10 melanoma cells stably expressing firefly luciferase were infected with lentiviruses carrying control shRNA or Jmjd6 shRNAs. These cells were implanted subcutaneously onto the right flank or injected intravenously into 6-week-old C57BL/6 mice. The growth/dissemination of B16F10 tumors was monitored weekly by bioluminescence imaging with the IVIS imaging system (Xenogen). Quantitative bioluminescent imaging was performed weekly after implantation or at 2.5 weeks after injection. A metastatic event was defined as any detectable luciferase signal above background. We found that, compared to that in control animals, the tumor size was much smaller and the tumor growth rate was significantly slower in mice received Jmjd6-depleted tumor cells (Fig. 4e). In addition, Jmjd6 depletion was associated with a marked suppression of the lung metastasis of B16F10 tumors, which were verified by bioluminescence imaging and histological staining (Fig. 4f). These experiments are consistent with in vitro observations and indicate that JMJD6 promotes the growth and metastasis of melanoma in vivo*.*

### JMJD6 enhances the Angiogenic potential of melanoma cells

**Fig. 5:**
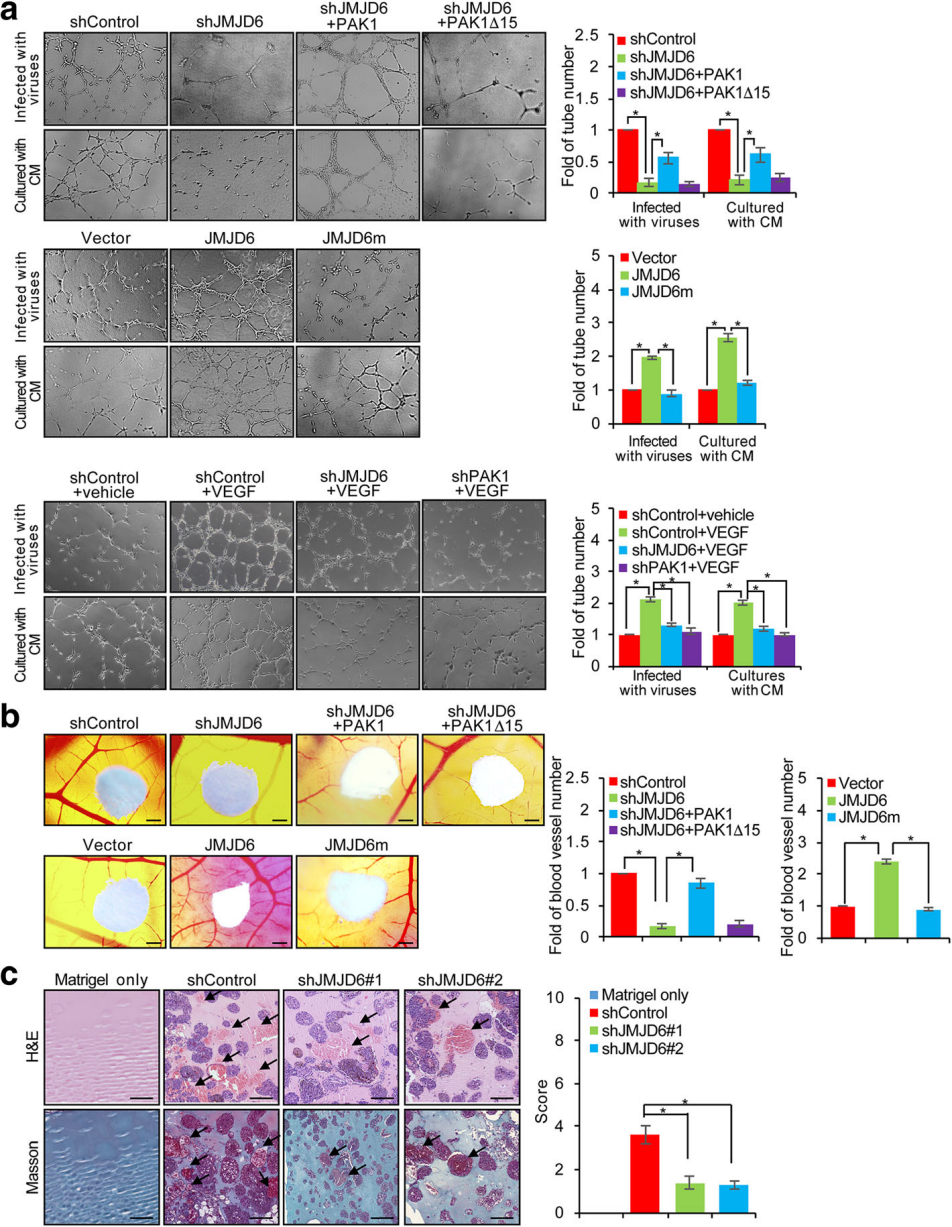
JMJD6 Enhances the Angiogenic Potential of Melanoma Cells. **a** A375 cells and HUVECs were infected with lentiviruses carrying control shRNA, JMJD6 shRNA or PAK1 shRNA, and/or infected with retroviruses carrying PAK1, PAK1Δ15, JMJD6, or JMJD6m. Then, the cells were added onto a solidified extracellular matrix and were treated with or without 20 ng/ml VEGF. After incubation, the number of endothelial tubes was counted under a light microscope. Representative images from each group were shown. Each bar represents the mean ± SD. Each independent experiment was performed at least three times. **b** A375 cells were infected with viruses carrying indicated constructs for YSM analysis. The number of blood vessels that migrated into the sponge was counted. Representative images from each group are shown. Each bar represents the mean ± SD. Scale bar = 250 μm. Each independent experiment was performed at least three times. **c** Matrigel only or Matrigel mixed with A375 cells infected with viruses carrying control shRNA or JMJD6 shRNAs were subcutaneously injected into 6-week-old BALB/c female mice (*n* = 6). The Matrigel plugs were removed for H&E and Masson trichrome staining. Scale bar = 200 μm. The relative photos are shown. Results were presented as the mean ± SD

It has been reported that the MAPK signaling pathway also influences angiogenesis [43–45]. To test that whether or not JMJD6 could also affect angiogenesis in melanoma, we first performed in vitro endothelial tube formation assays in which the ability of endothelial cells to form three-dimensional capillary-like tubular structures when cultured on extracellular matrix gels prepared from Engelbreth-Holm-Swarm tumor cells was assessed [46]. A375 cells and human umbilical vein endothelial cells (HUVECs) were infected with lentiviruses carrying control shRNA or JMJD6 shRNA, and/or infected with retroviruses carrying PAK1, PAK1Δ15, JMJD6, or JMJD6m. Conditional media (CM) from A375 cells were added onto solidified extracellular matrix gels. After incubation, the formation of endothelial cell tubes was examined under a light microscope, and the number of tubes was counted. The result revealed that JMJD6-depleted HUVECs or HUVECs cultured in CM from JMJD6-depleted A375 cells generated fewer endothelial tubes, compared to control, and simultaneous overexpression of PAK1, but not PAK1Δ15, could rescue this effect (Fig. 5a, top). More endothelial tubes were observed in A375 cells overexpressing JMJD6, whereas in cells overexpressing JMJD6m, the number of endothelial tubes was similar to that in control group (Fig. 5a, middle). Furthermore, we found that VEGF-stimulated HUVECs or HUVECs cultured in CM from vector-transfected A375 cells generated more endothelial tubes, while both JMJD6 knockdown and PAK1 knockdown led to a decrease in the formation of endothelial cell tubes compared to the control (Fig. 5a, bottom), suggesting that JMJD6 is capable of enhancing the angiogenic potential of HUVECs.

In vivo chicken yolk sac membrane (YSM) assays were then carried out in which gelatin sponges that had adsorbed suspensions of A375 cells infected with viruses carrying control shRNA or JMJD6 shRNA, and/or PAK1, PAK1Δ15, JMJD6, or JMJD6m were placed on top of the YSM. Microscopic analysis of the number of blood vessels entering the sponge showed that, compared to control, the eggs treated with JMJD6-depleted A375 cells formed significantly fewer blood vessels, an effect that could be effectively rescued by simultaneous overexpression of PAK1, but not PAK1Δ15, whereas overexpression of JMJD6, but not JMJD6m, resulted in an increase in blood vessels (Fig. 5b). The results support a role for JMJD6 in promoting blood vessel formation.

We next employed a well-established in vivo angiogenesis model, the Matrigel plug assay, to investigate the effect of JMJD6 on angiogenesis in vivo. In this assay, BALB/c female mice were randomly divided into four groups, and the animals in each group (*n* = 6) were subcutaneously injected with either Matrigel only or Matrigel containing A375 cells infected with lentiviruses carrying control shRNA or JMJD6 shRNAs. Seven days after injection, the mice were sacrificed, and the Matrigel plugs were processed and stained with H&E (hematoxylin and eosin) and Masson trichrome. Microscopic examination of Matrigel plugs revealed that, compared to that of control shRNA-treated Matrigels, only a few endothelial cells invaded the plugs of JMJD6 shRNAs-treated Matrigel (Fig. 5c). Taken together, the above results support a role for JMJD6 in promoting the angiogenesis in melanoma.

### JMJD6 is Transcriptionally activated by c-Jun

**Fig. 6:**
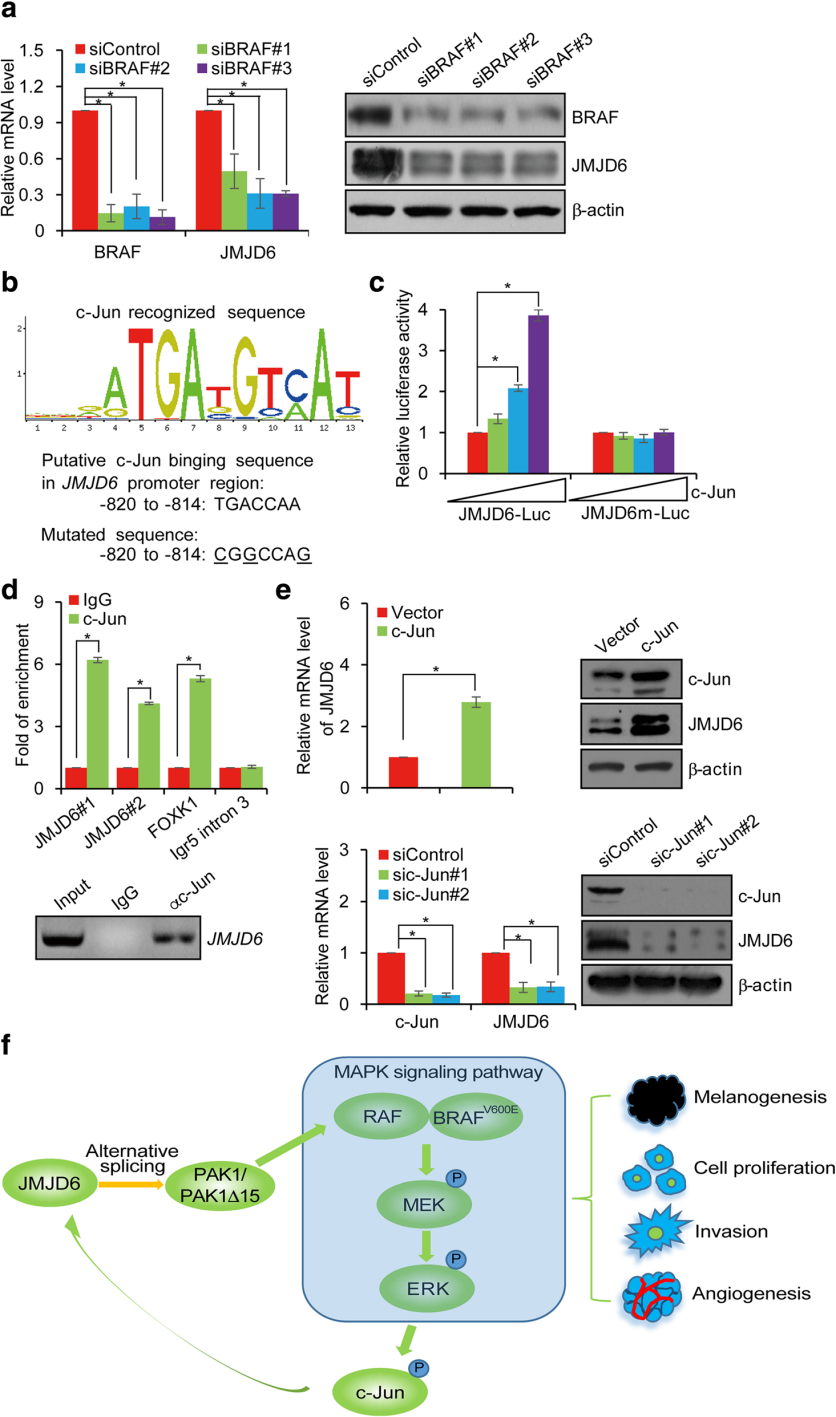
JMJD6 is Transcriptionally Activated by c-Jun. **a** A375 cells were transfected with control siRNA or BRAF siRNAs. Total RNAs and proteins were extracted and analyzed for the expressions of BRAF and JMJD6 by qRT-PCR and Western blotting, respectively. **b** c-Jun recognized consensus site was identified in the promoter region of *JMJD6* using a bioinformatics website (http://alggen.lsi.upc.es/cgi-bin/promo_v3/promo/promoinit.cgi?dirDB=TF_8.3). The number represents the nucleotide position relative to the transcription start site (+1). The mutated nucleotides are underlined. **c** Luciferase reporter assays in A375 cells transfected with JMJD6-Luc or mut-JMJD6-Luc together with c-Jun and renilla as indicated. **d** qChIP assays (upper) and ChIP assays (lower) of the occupancy of c-Jun in the promoter region of *JMJD6* in A375 cells. FOXK1 and Igr5 intron 3 serve as positive and negative control, respectively. **e** A375 cells were transfected with vector, c-Jun, or treated with control siRNA or c-Jun siRNAs. Total RNAs and proteins were extracted and analyzed for the expression of JMJD6 by qRT-PCR and Western blotting, respectively. **f** Proposed model of the JMJD6 in melanoma carcinogenesis. In melanoma cancer cells, JMJD6, through regulating PAK1 alternative splicing and positively influencing the MAPK signaling, promotes melanogenesis, cell proliferation, invasion and angiogenesis. Hyperactive MAPK signaling leads to the phosphorylation of c-Jun, which, in turn, transcriptionally activates JMJD6 expression. Such a self-enhancing molecular system favors the development and progression of melanoma

As described at the beginning, we found that JMJD6 was up-regulated in melanomas. Given our observations that JMJD6 influences, via regulation of PAK1 alternative splicing, multiple cellular processes in melanoma cells, understanding the aberrant regulation of JMJD6 in melanomas is of great significance. In this regard, it is worth noting that approximately 50% of melanomas harbor V600E mutation in BRAF, a genetic abnormality that renders the MAPK signaling pathway constitutively active. To investigate whether and how the hyperactive MAPK signaling contributes to the high expression of JMJD6, BRAF expression was knocked down in A375 cells. qRT-PCR and Western blotting analyses showed that both the mRNA and protein levels of JMJD6 in these cells were significantly reduced (Fig. 6a), suggesting that the MAPK signaling is functionally linked to JMJD6 expression, and that the MAPK signaling does so, though transcriptionally regulation.

Activation of the MAPK signaling leads to the phosphorylation of ERK, which, in turn, activates transcription factor c-Jun [6]. To investigate whether JMJD6 is a transcriptional target of c-Jun, we analyzed by bioinformatics the promoter of the *JMJD6* gene and found several putative c-Jun consensus binding sites. We thus cloned a 1157 bp fragment (−1057 to +100) from the *JMJD6* promoter containing the sequence TGACCAA (JMJD6-Luc) or containing a mutated form of this sequence (CGGCCAG, JMJD6m-Luc) in the upstream of a luciferase reporter gene (Fig. 6b) and co-transfected these constructs into A375 cells with c-Jun and renilla. The reporter assays revealed that c-Jun significantly stimulated the luciferase activity of JMJD6-Luc, but not that of JMJD6m-Luc (Fig. 6c). Chromatin immunoprecipitation (ChIP) assay confirmed the recruitment of c-Jun to the promoter region of *JMJD6* in A375 cells (Fig. 6d). Moreover, analyses by qRT-PCR and Western blotting in A375 cells transfected with vector or c-Jun or treated with control siRNA or c-Jun siRNAs showed that ectopic expression of c-Jun resulted in an increase of JMJD6 and knockdown of c-Jun resulted in a decrease of JMJD6 at both mRNA and protein levels (Fig. 6e). Together, these results support a notion that BRAF mutation results in constitutively active MAPK signaling, which, in turn, leads to transcriptional activation of JMJD6 by c-Jun. According to our working model, elevated JMJD6, through regulation of the alternative splicing of genes including that encodes for PAK1, promotes multiple cellular processes including melanogenesis, proliferation, invasion, and angiogenesis in melanoma cells, and eventually the development and progression of melanoma. According to this scheme, there appears existing a feedforward regulatory loop between JMJD6 and the MAPK signaling pathway in which genetic mutation of BRAF results in hyperactive MAPK signaling, which, in turn, leads to the activation of c-Jun. Activated c-Jun transactivates JMJD6, which, in turn, through regulation of the alternative splicing of PAK1, enhances the MAPK signaling and drives melanoma carcinogenesis.

## Discussion

The understanding of the cellular activity and biological function of the jumonji C domain-containing protein JMJD6 continues to evolve. This protein was initially reported as a cell surface protein that enhances the recruitment of phagocytic cells to sites of apoptosis. Subsequent studies describe JMJD6 as either a histone arginine demethylase that removes methyl moieties from histone molecules [12] or a lysine hydroxylase to target histone or non-histone proteins [14]. Specifically, we showed previously that JMJD6 acts as a lysyl hydroxylase to modulate p53 activity [13], and, recently, JMJD6 has been shown to hydroxylate RNA splicing factor U2AF65 and influence alternative splicing [14]. *JMJD6* null mice manifest early postnatal lethality, growth retardation, and multiple developmental abnormalities due to impaired differentiation during embryogenesis [15–17]. Clearly, JMJD6 exerts a multifaceted and important role in cell biology and animal development. Consistently, JMJD6 has been implicated in various pathological states including cancers [13, 18, 19]. We report in the current study that JMJD6 is up-regulated in a variety of cancers, including melanoma, thyroid cancer, ovarian cancer, breast cancer, prostate cancer, lung adenocarcinomas, liver cancer, and colorectal cancer. We found that the protein level of JMJD6 was significantly higher in melanomas than in normal tissues, and that high expression of JMJD6 was positively correlated with the clinicopathological stage, aggressiveness, and poor prognosis of melanoma.

To understand the mechanistic role of JMJD6 in melanoma carcinogenesis, we performed RNA-seq in melanoma cells and found that the alternative splicing of a panel of genes including that encoding for PAK1, a key component of the MAPK signaling pathway, is regulated by JMJD6. PAK1 acts to phosphorylate RAF and MEK [32–34], thereby positively regulating the MAPK signaling pathway. We showed that JMJD6 promotes the production of the full-length PAK1 and inhibits the generation of PAK1Δ15. Significantly, our experiments indicate that PAK1Δ15 represents a catalytically inactive isoform of PAK1 unable to phosphorylate RAF and MEK and to activate the MAPK signaling.

The MAPK signaling pathway is known to be critically implicated in the molecular pathogenesis of melanoma. Consistent with this, it has been reported that approximately 50% of melanomas carry an active mutation in *BRAF*, which renders the MAPK signaling pathway constitutively active [4]. Upon activation of the cascade, phosphorylation of the downstream effector kinases (ERK1/2) leads to transcriptional activation of cyclin D1, degradation of CDK inhibitor p27, and activation of p90 ribosomal S6 kinase (p90RSK), which in turn inactivates the cell cycle-inhibitory protein myelin transcription factor 1 (MYT1) [47, 48]. It is now abundantly clear that the MAPK signaling pathway represents a main cellular system controlling cell cycle progression and cell proliferation [47]. Thus, the regulation of PAK1 alternative splicing by JMJD6 is of significant importance to the MAPK signaling and cell proliferation. Indeed, we showed that ectopic expression of JMJD6 promoted the proliferation of melanoma cells, an effect that was dependent on the lysyl hydroxylase activity of JMJD6 and was through the regulation of the alternative splicing of PAK1.

In addition to cell proliferation, the MAPK pathway has also been implicated in the regulation of an array of cellular processes such as EMT, angiogenesis, and melanogenesis in melanoma. It is even reported that BRAF^V600E^ is a catalyst for EMT in thyroid carcinoma and melanoma [49]. Meanwhile, a recent study reported that JMJD6 promotes EMT in breast cancer [50]. In the current study, we demonstrated that JMJD6 promotes EMT and metastasis in melanoma, and we showed that JMJD6 does so, through its lysyl hydroxylase activity and via its regulation of the alternative splicing of PAK1. Our results provide a functional link between JMJD6 and the MAPK signaling pathway in the regulation of EMT and metastasis of melanoma. In support of the regulation of PAK1 thus the MAPK signaling by JMJD6, our experiments also revealed that JMJD6 regulates melanogenesis and angiogenesis in melanoma cells.

In the MAPK signaling cascade, activation of ERK1/2 leads to phosphorylation and activation of transcription factor c-Jun. Remarkably, we found in the current study that JMJD6 is transactivated by c-Jun. In light of our findings that JMJD6 is up-regulated in melanoma and the MAPK signaling is modulated by JMJD6 via regulation of the alternative splicing of PAK1 and the reported observation that a large proportion of melanoma carry BRAF^V600E^ mutation rendering the MAPK signaling constitutive active [4], there appears existing a feedforward regulatory loop between JMJD6 and the MAPK signaling pathway in which genetic mutation of BRAF results in hyperactive MAPK signaling, which, in turn, leads to the activation of c-Jun. Activated c-Jun transactivates JMJD6, which, in turn, through regulation of the alternative splicing of PAK1, enhances the MAPK signaling (Fig. 6f). Such a self-enhancing molecular system will surely favor the development and progression of melanoma.

## Conclusions

We report here that JMJD6, through regulating PAK1 alternative splicing and influencing the MAPK signaling, through affecting multiple aspects of melanoma cells, promotes melanoma carcinogenesis, supporting the pursuit of JMJD6 as a potential prognostic biomarker and a therapeutic target for melanoma.

## Additional file

Additional file 1: Table S1. Correlation between JMJD6 expression and clinicopathologic characteristics of 88 melanoma patients by chi-square test. (DOCX 503 kb)
Additional file 2: Table S2. Alternative splicing events in MAPK pathway. (DOCX 19 kb)

## Abbreviations

EMT: Epithelial-to-mesenchymal transition
JMJD6: JmjC domain-containing protein 6
MAPK: Mitogen-activated protein kinase
PAK1: p21 (RAC1) activated kinase 1
