# Supplementary material for: JMJD6 promotes melanoma carcinogenesis through regulation of the alternative splicing of PAK1, a key MAPK signaling component
Source: Mol Cancer. 2017 Nov 29;16:175. doi: 10.1186/s12943-017-0744-2 (PMC5708181; doi:10.1186/s12943-017-0744-2)
Supplement: Additional file 1: Table S1. — Correlation between JMJD6 expression and clinicopathologic characteristics of 88 melanoma patients by chi-square test. (DOCX 503 kb) [file 12943_2017_744_MOESM1_ESM.docx]

Table S1. Correlation between JMJD6 expression and clinicopathologic characteristics of 88 melanoma patients by chi-square test

| Variables | n | JMJD6 expression | | *P value* |
| --- | --- | --- | --- | --- |
|  |  | Negative (n, %) | Positive (n, %) |  |
| *Gender* |  |  |  |  |
| Male | 52 | 29 (54.72%) | 23 (65.71%) | *p=0.304* |
| Female | 36 | 24 (45.28%) | 12 (34.29%) |  |
| *Age (years)* |  |  |  |  |
| <50 | 34 | 24 (45.28%) | 10 (28.57%) | *p=0.115* |
| ≥50 | 54 | 29 (54.72%) | 25 (71.43%) |  |
| *TN**M stage* |  |  |  |  |
| I-II | 77 | 50 (94.34%) | 27 (77.14%) | *p=0.017* |
| III-IV | 11 | 3 (5.66%) | 8 (22.86%) |  |
| *Depth of invasion* |  |  |  |  |
| 1-2 | 24 | 14 (26.42%) | 10 (28.57%) | *p=0.824* |
| 3-4 | 64 | 39 (73.58%) | 25 (71.43%) |  |
| *Lymph node* |  |  |  |  |
| N0 | 80 | 51 (96.23%) | 29 (82.86%) | *p=0.033* |
| N1-N2 | 8 | 2 (3.77%) | 6 (17.14%) |  |
| *Distant metastasis* |  |  |  |  |
| M0 | 42 | 30 (56.60%) | 12 (34.29%) | *p=0.040* |
| M1 | 46 | 23 (43.40%) | 23 (65.71%) |  |
