# Supplementary material for: JMJD6 promotes melanoma carcinogenesis through regulation of the alternative splicing of PAK1, a key MAPK signaling component
Source: Mol Cancer. 2017 Nov 29;16:175. doi: 10.1186/s12943-017-0744-2 (PMC5708181; doi:10.1186/s12943-017-0744-2)
Supplement: Additional file 2: Table S2. — Alternative splicing events in MAPK pathway. (DOCX 19 kb) [file 12943_2017_744_MOESM2_ESM.docx]

Table S2. Alternative splicing events in MAPK pathway

| Gene symbol | Location | Exon Number | Splicing Type | Description |
| --- | --- | --- | --- | --- |
| *PAK1* | chr11:77036373-77036421 | 15 | Cassette exon | p21 (RAC1) activated kinase 1 |
| *RAPGEF2* | chr4:160272285-160272333 | 29 | Alternative 5’ splice site | cDNA FLJ61094, highly similar to Rap guanine nucleotide exchange factor 2 |
| *MAP3K4* | chr6:161514871-161514883 | 15 | Alternative 5’ splice site | Mitogen-activated protein kinase kinase kinase 4 |
| *FLNB* | chr3:58127623-58127656 | 32 | Alternative 5’ splice site | Filamin B, beta (Actin binding protein 278) |
| *CACNB3* | chr12:49210656-49210778 | 3 | Cassette exon | cDNA FLJ58949, highly similar to Voltage-dependent L-type calcium channel subunit beta-3 |
| *TGFBR1* | chr9:101894779-101894791 | 3 | Alternative 3’ splice site | Transforming growth factor beta receptor 1 |
| *NFATC4* | chr14:24845760-24846084 | 14 | Alternative 5’ splice site | cDNA FLJ55193, highly similar to Nuclear factor of activated T-cells, cytoplasmic 4 |
| *TGFBR2* | chr3:30664691-30664765 | 2 | Cassette exon | Transforming growth factor beta receptor 2 |
| *GNG12* | chr1:68297502-68297653 | 2 | Cassette exon | Guanine nucleotide-binding protein subunit gamma |
| *CASP3* | chr4:185569619-185569785 | 2 | Cassette exon | Caspase-3 |
